# Human Poisoning with Methomyl and Cypermethrin Pesticide Mixture

**DOI:** 10.3390/toxics11040372

**Published:** 2023-04-14

**Authors:** Chi-Ang Liang, Shu-Sen Chang, Hsien-Yi Chen, Kai-Fan Tsai, Wen-Chin Lee, I-Kuan Wang, Chao-Yu Chen, Shou-Hsuan Liu, Cheng-Hao Weng, Wen-Hung Huang, Ching-Wei Hsu, Tzung-Hai Yen

**Affiliations:** 1 Department of Nephrology, Clinical Poison Center, Chang Gung Memorial Hospital, Linkou Branch and College of Medicine, Chang Gung University, Taoyuan 333, Taiwan; 2 Institute of Health Behaviors and Community Sciences, Department of Public Health, College of Public Health, National Taiwan University, Taipei 100, Taiwan; 3 Department of Emergency Medicine, Chang Gung Memorial Hospital, Linkou Branch and College of Medicine, Chang Gung University, Taoyuan 333, Taiwan; 4 Division of Nephrology, Department of Internal Medicine, Kaohsiung Chang Gung Memorial Hospital, Chang Gung University College of Medicine, Kaohsiung 833, Taiwan; 5 Department of Nephrology, China Medical University Hospital and College of Medicine, China Medical University, Taichung 406, Taiwan

**Keywords:** methomyl, cypermethrin, pesticide mixture, poisoning, acute respiratory failure, mortality

## Abstract

There is limited literature analyzing the outcome of human poisoning with methomyl and cypermethrin pesticide mixture. Between 2002 and 2018, a total of 63 patients intoxicated with methomyl, cypermethrin, or their pesticide mixture were treated at Chang Gung Memorial Hospital. The patients were categorized into three groups based on the type of pesticide, as methomyl (n = 10), cypermethrin (n = 31), or methomyl and cypermethrin (n = 22). Demographic, clinical, laboratory, and mortality data were obtained for analysis. The patients were aged 54.9 ± 18.9 years. Following ingestion, the patients experienced a wide range of clinical symptoms, including aspiration pneumonia (50.8%), acute respiratory failure (41.3%), acute kidney injury (33.3%), multiple organ failure (19.0%), emesis (19.0%), acute hepatitis (12.7%), diarrhea (7.9%), seizures (4.8%), lacrimation (4.8%), etc. After analysis, it was found that patients with methomyl and cypermethrin poisoning suffered higher incidences of acute respiratory failure (*p* < 0.001), aspiration pneumonia (*p* = 0.004), acute kidney injury (*p* = 0.011), and multiple organ failure (*p* < 0.001) than the other groups. Laboratory analyses revealed that patients with methomyl and cypermethrin poisoning had a higher creatinine level (*p* = 0.011), white blood cell count (*p* < 0.001), and neutrophil count (*p* = 0.019) than the other groups. A total of seven (11.1%) patients died. The average duration of hospitalization was 9.8 ± 10.0 days. In a multivariate logistic regression model, it was revealed that methomyl pesticide (*p* = 0.045) or methomyl and cypermethrin pesticide mixture (*p* = 0.013) were significant risk factors for acute respiratory failure. Nevertheless, no mortality risk factor could be identified. Therefore, the analytical results suggest that methomyl pesticide is the major contributor to the toxicity of methomyl and cypermethrin pesticide mixture poisoning. More research is needed.

## 1. Introduction

The pesticide mixture of methomyl and cypermethrin is a combination of two different types of pesticides, carbamate and pyrethroid. Methomyl is an anti-cholinesterase carbamate pesticide, whereas cypermethrin is a synthetic pyrethroid insecticide. The use of mixtures of insecticides with different modes of action has become common practice in pest control in order to increase the effectiveness of the insecticide and reduce the risk of developing resistance. 

Cypermethrin can enhance neuronal excitation by prolonging the activation of sodium channels. In humans, large-dose exposure to cypermethrin can result in a range of symptoms, including tingling or numbness (paresthesia), excessive salivation, nausea, vomiting, dizziness, muscle twitching (fasciculation), altered mental status, coma, seizures, and acute lung injury [1]. Carbamate works by reversibly inhibiting various types of esterase. The primary mechanism of their toxicity is the inhibition of acetylcholinesterase, which leads to excessive cholinergic overstimulation [2]. The symptoms of carbamate poisoning include excessive salivation and tearing, muscle twitching and weakness, constricted pupils, decreased levels of consciousness, respiratory failure, and seizures.

The maximum residue level (MRL) for methomyl varies between different crops, ranging from 0.02 to 20 mg/kg, depending on the sensitivity of the plant and the amount of consumption by humans. In the United States, the Environmental Protection Agency categorizes methomyl under toxicity category I, indicating high toxicity and severe irritation via the oral route. As a restricted use pesticide, methomyl can only be used by or under the direct supervision of specially trained and certified applicators. Methomyl is no longer an approved active substance, according to the European Union pesticide regulation [3]. Taiwan prohibited the use of 90% methomyl water soluble granules and 90% methomyl wet table powder in 2006, as well as 24% methomyl solution in 2017. However, the pesticide mixture of methomyl and cypermethrin (with an effective concentration of 13.5%) was exempted from the prohibition.

Given the limited research on the toxicity and potential harm to human health of the pesticide mixture of methomyl and cypermethrin, the objective of this study was to conduct research to determine whether poisoning from this compound is as severe as, or even more severe than, the poisoning caused by individual methomyl or cypermethrin compounds. The analytical results could provide evidence of the nature of poisoning caused by the pesticide mixture of methomyl and cypermethrin, and could provide more toxicity information for local health authorities.

## 2. Materials and Methods

### 2.1. Patients 

Between 2002 and 2018, a total of 63 patients intoxicated with methomyl, cypermethrin, or their pesticide mixture were treated at Chang Gung Memorial Hospital. The patients were categorized into three groups based on the type of pesticide, as methomyl (n = 10), cypermethrin (n = 31), or methomyl and cypermethrin (n = 22). All the patients were exposed via oral ingestion. Demographic, clinical, laboratory, and mortality data were obtained for analysis. 

### 2.2. Inclusion and Exclusion Criteria 

All the patients were included in this analysis. Patients were excluded if they had ingested pesticides other than methomyl or cypermethrin, or if their exposure was not oral. 

### 2.3. Clinical Diagnosis of Pesticide Poisoning

The diagnosis was based on exposure history, clinical manifestation, physical findings, and laboratory results. Serum cholinesterase activity was determined using enzymatic method DF51 (Siemens Healthcare Diagnostics, Newark, DE, USA). The normal reference range was 7–19 U/mL, with a lower limit of quantification of 0.8 U/mL. As serum cholinesterase activity is not specific to methomyl, a comprehensive clinical history was taken, which involved questioning the patient and family about the pesticide label picture and requesting the pesticide bottle for identification.

### 2.4. Clinical Management 

Patients with pesticide poisoning received gastric lavage with a 2-L solution of 0.9% normal saline, followed by the administration of activated charcoal via a nasogastric tube. Gastric lavage was considered if the patient presented within 1 h after oral ingestion. The contraindications comprise loss of airway reflexes, ingestion of a strong acid or alkali, or the risk of gastrointestinal hemorrhage due to an underlying disorder [4]. Activated charcoal was used for the prevention of further absorption. Methomyl pesticide or methomyl and cypermethrin pesticide mixture poisoned patients with depressed serum cholinesterase levels were given appropriate antidotes, including anti-cholinergic and oxime medications. The treatment for bronchial secretions and bronchospasms consisted of intravenous atropine at a starting dose of 2 mg, which was titrated as necessary. Pralidoxime therapy, at a dose of 1 g every 4 h, was also administered to patients experiencing cholinergic crisis. The indication for an antidote was based on depressed serum cholinesterase levels and cholinergic crisis symptoms. There is no antidote available for cypermethrin poisoning, and its management is mainly symptomatic and supportive.

### 2.5. Statistical Analysis 

In this study, we presented continuous variables as the mean and standard deviation and reported categorical variables as frequencies with percentages. Comparisons of variables among the three pesticide groups were performed using trend estimation. A one-way analysis of variance was used when assessing for differences in one continuous variable between the three groups. A univariate binary logistic regression analysis was performed to analyze the potential variables that may be associated with acute respiratory failure or mortality. In order to control for the confounders, stepwise backward multivariate binary logistic regression was performed to analyze variables that were found to be significant (*p* value < 0.05) in the univariate analysis. A *p* value of less than 0.05 was designated as the significance threshold to reject the null hypothesis. All the analyses were performed using IBM SPSS Statistics version 20.0 (IBM Corp., Armonk, NY, USA).

## 3. Results

A significant trend comparison of the baseline characteristics of patients with pesticide poisoning is outlined in Table 1. The patients were aged 54.9 ± 18.6 years and almost half of the patients were male (49.2%). More men ingested the pesticide by intention than women (56.6% versus 43.4%, *p* = 0.043), but no age difference was noted between the intentional and unintentional groups (54.0 versus 59.4 years, *p* = 0.406). Most of the cases of unintentional pesticide exposure were due to the unintentional ingestion of pesticides that had been stored in drinking water bottles. The time between pesticide ingestion and hospital arrival was 2.6 ± 1.7 h. The occupations of the patients were non-farmers (55.6%) and unemployed (36.5%), and there were fewer farmers (7.9%). After the analysis, it was found that the patients with methomyl and cypermethrin pesticide mixture poisoning had a greater time interval between ingestion and arrival at the hospital (*p* < 0.001) and a higher Poisoning Severity Score (*p* < 0.001), but a lesser smoking history (*p* = 0.036) than the other groups. No significant differences were observed for the other baseline variables.

Following ingestion (Table 2), the patients experienced a wide range of clinical symptoms, including aspiration pneumonia (50.8%), acute respiratory failure (41.3%), acute kidney injury (33.3%), multiple organ failure (19.0%), emesis (19.0%), acute hepatitis (12.7%), diarrhea (7.9%), seizures (4.8%), lacrimation (4.8%), etc. Notably, patients with methomyl and cypermethrin pesticide mixture poisoning suffered higher incidences of acute respiratory failure (*p* < 0.001), aspiration pneumonia (*p* = 0.004), acute kidney injury (*p* = 0.011), and multiple organ failure (*p* < 0.001) than the other groups. No significant differences were observed for the other clinical variables.

The laboratory analysis (Table 3) revealed that the patients with methomyl and cypermethrin pesticide mixture poisoning had higher creatinine levels (*p* = 0.011), white blood cell counts (*p* < 0.001), and neutrophil counts (*p* = 0.019) than the other groups.

As shown in Table 4, a total of seven (11.1%) patients died despite intensive treatment. The average duration of hospitalization was 9.8 ± 10.0 days, and the average duration of intensive care unit hospitalization was 4.2 ± 7.9 days.

The univariate analysis revealed that methomyl poisoning (*p* < 0.001), methomyl and cypermethrin pesticide mixture poisoning (*p* < 0.001), aspiration pneumonia (*p* < 0.001), acute kidney injury (*p* < 0.001), and white blood cell count (*p* < 0.001) were significant risk factors for acute respiratory failure. In a multivariate logistic regression model (Table 5), it was confirmed that methomyl pesticide poisoning (*p* = 0.045) and methomyl and cypermethrin pesticide mixture poisoning (*p* = 0.013) were significant risk factors for acute respiratory failure. Nevertheless, no mortality risk factors could be identified.

## 4. Discussion

There is limited literature analyzing the outcome of human poisoning with methomyl and cypermethrin pesticide. In 1977, unintended methomyl ingestion was first demonstrated to result in severe intoxication, which presented with seizures and bronchospasms [5]. A review of the literature revealed that poisoning with methomyl induces severer effects than poisoning with cypermethrin (Table 6). A retrospective observational study conducted in Spain in 1990 found that methomyl rarely causes serious complications [6]. However, subsequent studies in Korea and Tunisia reported a considerable proportion of acute respiratory failure (17.0–76.5%) and mortality (13.5–17.6%) after methomyl poisoning [7,8]. On the other hand, studies in Taiwan, Korea, and India found a relatively low incidence of acute respiratory failure (0–17.9%) and mortality (0–3.6%) after cypermethrin poisoning [9,10,11]. Notably, Hu et al. described one rare case report in which a patient exhibited acute cholinergic crisis, cortical blindness, and delayed peripheral neuropathy after methomyl-alphamethrin pesticide mixture ingestion [12]. 

The incidence rates of acute respiratory failure in the methomyl and cypermethrin group, the methomyl group, and the cypermethrin group were 72.7%, 70.0%, and 9.7%, respectively (Table 2). Furthermore, a multivariate logistic regression analysis disclosed that methomyl pesticide poisoning (*p* = 0.045) or methomyl and cypermethrin pesticide mixture poisoning (*p* = 0.013) were significant risk factors for acute respiratory failure (Table 5). This observation is consistent with previous reports that have linked methomyl poisoning to acute respiratory failure [7,8]. In research related to organophosphates, which are anti-cholinesterase agents that are similar to methomyl, the mechanism leading to respiratory failure can be divided into two main categories: central apnea and pulmonary dysfunction. These categories include the disruption of rhythmic activities associated with respiration in the brainstem and the inhibition of pulmonary secretory, airway, and vascular function in lung tissue [13]. Exposure to pyrethroids has been linked to various detrimental effects, including oxidative stress, inflammation, and DNA damage. These pathophysiological mechanisms may be associated with the development of acute lung injury [14]. 

As shown in Table 2, aspiration pneumonia was common in the methomyl and cypermethrin group (77.3%) and the methomyl group (50%), followed by the cypermethrin group (32.3%). Methomyl is known to inhibit the activity of acetylcholinesterase, resulting in acute cholinergic crisis and subsequent respiratory failure [15,16], which may lead to unconsciousness accompanied by various symptoms of muscarinic receptor stimulation, such as bronchospasms and vomiting. Concurrently, nicotinic receptor stimulation results in neuromuscular weakness and paralysis, which could elevate the risk of the aspiration of gastric contents [16]. 

Table 2 shows that acute kidney injury was common in the methomyl and cypermethrin group (54.5%) and the methomyl group (40.0%), followed by the cypermethrin group (16.1%). The pathogenesis of methomyl-induced kidney injury is not well understood, but possible mechanisms, similar to those of organophosphate toxicity, include direct damage to the renal tubules, oxidative stress, rhabdomyolysis, and hypovolemic status associated with dehydration [17]. It is important to highlight that previous studies have established a relationship between acute kidney injury and acute lung injury. The latter results in the release of inflammatory mediators into the bloodstream due to lung damage, which can adversely affect renal function [18,19]. Interestingly, in a meta-analysis study, Dutch researchers revealed that endotracheal intubation is associated with a threefold increase in the odds of developing acute kidney injury [20]. 

The patients with methomyl poisoning suffered lower systolic (*p* = 0.005) and diastolic (*p* = 0.045) pressures than the other groups (Table 2). There was no clear explanation for this. Nevertheless, the blood pressure variable was not a significant predictor of mortality after multivariate logistic regression analysis.

The intentional ingestion of pesticides is common in Taiwan because of easy access [21,22]. In this study, it was found that the occupation of farming was relatively uncommon (methomyl group, 0%; cypermethrin group, 3.2%; methomyl and cypermethrin group, 18.2%). In contrast, the proportion of intentional poisoning was relatively high (methomyl group, 60.0%; cypermethrin group, 90.3%; methomyl and cypermethrin group, 86.4%). The results of this study are in line with previous research on pesticide poisoning in Taiwan, which found that the proportion of pesticide poisoning among farmers (20–32.4%) was lower compared to non-farmers (67.6–80.0%) [23,24]. This implies that non-agricultural persons have easy access to pesticides and can obtain pesticides for self-poisoning. In Taiwan, toxic pesticides are readily accessible in farming households. People can easily obtain pesticides and most pesticides are unlocked. Therefore, it is suggested that the government should take action to ensure safe access to pesticides for suicide prevention.

Table 1 shows that intentional ingestion accounted for 84.1% of cases, but only seven patients died (11.1%). It is unclear whether the suicide attempt was just a cry for help, as the patients may have been unaware of the toxicity of these pesticides. Theoretically, the majority of patients who engage in suicidal behavior are hesitant about desiring to die at the moment of the act, and some suicidal attempts are impulsive reactions to acute stressors [25]. This data could possibly be explained by the policy implemented by the Taiwan government involving prohibiting the sale of pesticides that are most lethal to humans after ingestion, as well as subsidizing the cost of pesticides that are less toxic to humans.

The retrospective nature of the study, small sample size, lack of information on blood methomyl and cypermethrin concentrations, lack of information on petroleum residues in thw pesticides, lack of information on non-oral routes of exposure, lack of information on other pesticides, and a short follow-up duration limit the certainty of our conclusions. More research is needed in order to fully understand the outcomes of methomyl and cypermethrin pesticide mixture poisoning.

## 5. Conclusions

The data presented in this study are significant as it is the only original study conducted on patients with methomyl and cypermethrin pesticide mixture poisoning. The analytical results suggest that methomyl pesticide poisoning accounted for the toxicity of methomyl and cypermethrin pesticide mixture poisoning.

## Figures and Tables

**Table 1:** Baseline characteristics of patients with pesticide poisoning, stratified by type of pesticide (n = 63).

Variable	All Patients (n = 63)	Patients with Methomyl Poisoning (n = 10)	Patients with Cypermethrin Poisoning (n = 31)	Patients with Methomyl and Cypermethrin Poisoning (n = 22)	*p* Value
Age, year	54.9 ± 18.6	44.9 ± 21.8	58.0 ± 17.5	55.1 ± 17.7	0.155
Male, n (%)	31 (49.2)	7 (70.0)	15 (48.4)	9 (40.9)	0.310
Time between ingestion and hospital arrival (h)	2.6 ± 1.7	3.4 ± 1.3	1.8 ± 1.2	3.7 ± 1.8	<0.001 ***
Occupation					
Farmer, n (%)	5 (7.9)	0 (0)	1 (3.2)	4 (18.2)	
Non-farmer, n (%)	35 (55.6)	7 (70)	14 (45.2)	14 (63.6)	
Unemployed, n (%)	23 (36.5)	3 (30)	16 (51.6)	4 (18.2)	
Intentional ingestion, n (%)	53 (84.1)	6 (60.0)	28 (90.3)	19 (86.4)	0.070
Ingested amount (mL)	144.1 ± 151.3	234.0 ± 184.3	145.8 ± 169.0	106.2 ± 78.8	0.280
Hypertension, n (%)	14 (22.2)	1 (10.0)	8 (25.8)	5 (22.7)	0.590
Diabetes mellitus, n (%)	9 (14.3)	1 (10.0)	6 (19.4)	2 (9.1)	0.539
Smoking habit, n (%)	14 (22.2)	5 (50.0)	7 (22.6)	2 (9.1)	0.036 *
Alcoholic consumption habit, n (%)	19 (30.2)	5 (50.0)	8 (25.8)	6 (27.3)	0.338
Charlson comorbidity index	2.1 ± 2.2	1.2 ± 2.1	2.5 ± 2.4	1.9 ± 1.9	0.221
Poisoning Severity Score	2.2 ± 0.2	2.7 ± 0.6	1.7 ± 0.3	2.9 ± 0.3	<0.001 ***

Note: * *p* < 0.05, *** *p* < 0.001.

**Table 2:** Clinical findings of patients with pesticide poisoning, stratified by type of pesticide (n = 63).

Variable	All Patients (n = 63)	Patients with Methomyl Poisoning (n = 10)	Patients with Cypermethrin Poisoning (n = 31)	Patients with Methomyl and Cypermethrin Poisoning (n = 22)	*p* Value
Respiratory system					
Aspiration pneumonia, n (%)	32 (50.8)	5 (50.0)	10 (32.3)	17 (77.3)	0.004 **
Acute respiratory failure, n (%)	26 (41.3)	7 (70.0)	3 (9.7)	16 (72.7)	<0.001 ***
Cardiovascular system					
Systolic blood pressure, mmHg	137.1 ± 38.7	102.0 ± 62.7	146.3 ± 25.0	140.3 ± 33.1	0.005 *
Diastolic blood pressure, mmHg	80.2 ± 23.9	67.7 ± 39.4	86.0 ± 15.5	79.1 ± 22.4	0.045 *
Heart rate, beats per minute	96.2 ± 30.3	79.0 ± 46.3	93.9 ± 22.6	107.2 ± 28.0	0.040 *
Corrected QT-interval prolongation, n (%)	1 (1.6)	1 (11.1)	0	0	0.050
Gastrointestinal system					
Emesis, n (%)	12 (19.0)	1 (10.0)	7 (22.6)	4 (18.2)	0.684
Diarrhea, n (%)	5 (7.9)	1 (10.0)	4 (12.9)	0 (0)	0.231
Acute hepatitis, n (%)	8 (12.7)	2 (20.0)	1 (3.2)	5 (22.7)	0.084
Neurological system					
Seizure, n (%)	3 (4.8)	0 (0)	0 (0)	3 (13.6)	0.053
Lacrimation, n (%)	3 (4.8)	1 (10.0)	1 (3.2)	1 (4.5)	0.692
Genitourinary system					
Acute kidney injury, n (%)	21 (33.3)	4 (40.0)	5 (16.1)	12 (54.5)	0.011 *
Others					
Multiple organ failure, n (%)	12 (19.0)	1 (10.0)	1 (3.2)	10 (45.5)	<0.001 ***

Note: The laboratory data were collected upon admission. * *p* < 0.05, ** *p* < 0.01, *** *p* < 0.001.

**Table 3:** Laboratory data of patients with pesticide poisoning, stratified by type of pesticide (n = 63).

Variable	All Patients (n = 63)	Patients with Methomyl Poisoning (n = 10)	Patients with Cypermethrin Poisoning (n = 31)	Patients with Methomyl and Cypermethrin Poisoning (n = 22)	*p* Value
Serum cholinesterase, U/mL	5.4 ± 6.0	3.2 ± 2.3	9.4 ± 7.3	3.2 ± 4.0	0.002 *
Blood urea nitrogen, mg/dL	15.0 ± 8.4	15.9 ± 9.4	13.2 ± 7.5	16.8 ± 8.9	0.383
Creatinine, mg/dL	1.1 ± 0.4	1.1 ± 0.3	0.9 ± 0.3	1.3 ± 0.5	0.011 *
Alanine transaminase, U/L	48.5 ± 66.7	77.8 ± 134.9	28.1 ± 20.8	62.8 ± 58.5	0.073
Sodium, mEq/L	140.0 ± 3.6	142.3 ± 4.8	139.7 ± 3.5	139.4 ± 2.9	0.107
Potassium, mEq/L	3.6 ± 0.6	3.6 ± 0.5	3.6 ± 0.5	3.6 ± 0.7	0.972
White blood cell count, 1000/μL	15.6 ± 7.3	19.0 ± 6.2	11.1 ± 5.5	20.2 ± 6.4	<0.001 ***
Neutrophil, %	73.2 ± 23.5	80.3 ± 17.0	65.2 ± 28.6	82.6 ± 9.0	0.019 *
Lymphocyte, %	14.4 ± 12.2	13.7 ± 16.1	16.4 ± 13.2	11.7 ± 7.8	0.415
Monocyte, %	4.4 ± 2.5	5.3 ± 2.3	4.2 ± 2.6	4.3 ± 2.4	0.500
Eosinophil, %	0.7 ± 1.3	0.3 ± 0.7	0.9 ± 1.3	0.5 ± 1.6	0.513
Basophil, %	1.2 ± 7.7	0.1 ± 0.2	0.2 ± 0.3	3.3 ± 13.4	0.336
Red blood cell count, million/μL	4.7 ± 0.7	5.0 ± 0.5	4.6 ± 0.7	4.7 ± 0.8	0.225
Hemoglobin, g/dL	14.0 ± 2.5	15.0 ± 1.6	13.5 ± 2.6	14.3 ± 2.6	0.260
Mean corpuscular volume, fL	89.4 ± 8.6	89.6 ± 8.4	88.2 ± 9.4	90.8 ± 7.6	0.584
Mean corpuscular hemoglobin, pg/Cell	30.0 ± 3.7	30.0 ± 3.3	29.5 ± 4.2	30.1 ± 3.4	0.658
Mean corpuscular hemoglobin concentration, gHb/dL	33.5 ± 1.9	33.5 ± 1.9	33.4 ± 2.0	33.6 ± 1.8	0.923
Red blood cell volume distribution width, %	14.0 ± 1.9	14.2 ± 1.7	14.1 ± 1.8	13.8 ± 2.1	0.817
Platelet, 1000/μL	251.4 ± 73.3	267.0 ± 69.9	242.0 ± 85.4	257.1 ± 56.9	0.625

Note: The laboratory data were collected upon hospital arrival. * *p* < 0.05, *** *p* < 0.001.

**Table 4:** Outcomes of patients with pesticide poisoning, stratified by type of pesticide (n = 63).

Variable	All Patients (n = 63)	Patients with Methomyl Poisoning (n = 10)	Patients with Cypermethrin Poisoning (n = 31)	Patients with Methomyl and Cypermethrin Poisoning (n = 22)	*p* Value
Mortality, n (%)	7 (11.1)	1 (10.0)	2 (6.5)	4 (18.2)	0.418
Oxime therapy, n (%)	30 (47.6)	9 (90.0)	0 (0)	21 (95.5)	0.001 ***
Duration of hospitalization, day	9.8 ± 10.0	9.7 ± 9.2	9.1 ± 10.2	10.8 ± 10.3	0.844
Duration of intensive care unit hospitalization, day	4.2 ± 7.9	4.7 ± 5.1	3.0 ± 8.3	5.7 ± 8.3	0.479

Note: *** *p* < 0.001.

**Table 5:** Analysis of acute respiratory failure using a multivariate logistic regression model (n = 63).

Variable	Univariate Analysis	Multivariate Analysis
	*p* Value	*p* Value
Patients with methomyl poisoning (as a reference)	<0.001 ***	0.045 *
Patients with cypermethrin poisoning	0.874	0.998
Patients with methomyl and cypermethrin poisoning	<0.001 ***	0.013 *
Aspiration pneumonia (yes)	<0.001 ***	0.998
Acute kidney injury (yes)	<0.001 ***	0.299
White blood cell count (per 1000/μL increase)	<0.001 ***	0.946

Note: * *p* < 0.05. *** *p* < 0.001.

**Table 6:** Published original studies of human poisoning with methomyl and cypermethrin pesticides.

Study	Year	Area	Sample Size	Pesticide	Respiratory Failure Rate, %	Mortality Rate, %
Present study	2023	Taiwan	10	Methomyl	70.0	10
			31	cypermethrin	9.7	6.5
			22	Methomyl and cypermethrin	72.7	18.2
Chaouali et al. [8]	2014	Tunisia	52	Methomyl	17.0	13.5
Iyyadurai et al. [10]	2014	India	32	Cypermethrin	0	0
Cha et al. [9]	2013	Korea	56	Pyrethroid (27.5% cypermethrin)	17.9	3.6
Lee et al. [7]	2011	Korea	17	Methomyl	76.5	17.6
Yang et al. [11]	2002	Taiwan	48	Cypermethrin	12.5	2.1
Martinez-Chuecos et al. [6]	1990	Spain	11	Methomyl	0	0

## Data Availability

The datasets used and analyzed for this study are available from the corresponding author upon request.
